# Targeting necroptosis: how does exercise protect against metabolic dysfunction-associated fatty liver disease?

**DOI:** 10.3389/fphys.2026.1760364

**Published:** 2026-05-15

**Authors:** Wenjing Song, Zhifei Ke

**Affiliations:** 1 School of Physical Education, Xihua University, Chengdu, China; 2 School of Physical Education, Sichuan University, Chengdu, China

**Keywords:** metabolic dysfunction-associated steatotic liver disease, necroptosis, exercise, mechanisms, RIPK1/RIPK3

## Abstract

Metabolic dysfunction-associated steatotic liver disease (MASLD) is a systemic metabolic disease that poses a serious threat to global health. If left untreated, it may progress to metabolic dysfunction-associated steatohepatitis (MASH), ultimately leading to liver fibrosis, cirrhosis, and hepatocellular carcinoma. Clarifying the underlying mechanisms of MASLD is therefore essential for both prevention and treatment. Although the pathophysiology of MASLD still remain incompletely understood, accumulating evidence supports a role for necroptosis in its development. In parallel, exercise is well established to improve metabolic dysfunction, hepatic steatosis, and inflammation associated with MASLD. Emerging studies further suggest that exercise can modulate signaling pathways related to necroptosis in various tissues. However, it should be emphasized that direct experimental evidence demonstrating that exercise alleviates MASLD specifically through regulating of hepatic necroptosis is currently lacking. Most of the proposed links between exercise and necroptosis in the context of MASLD are therefore based on indirect evidence derived from non-hepatic models or from independent lines of research and should be considered biologically plausible but not yet causally established mechanisms. In this review, we summarize current evidence supporting the involvement of necroptosis in MASLD, as well as evidence for the metabolic and anti-inflammatory effects of exercise. We then discuss potential mechanisms—including the RIPK1/RIPK3, AMPK/mTOR, and cGAS/STING pathways—through which exercise may influence MASLD in relation to necroptosis, while clearly distinguishing between evidence-supported findings and hypothesis-driven interpretations. Overall, this review proposes a conceptual framework linking exercise, necroptosis, and MASLD, but also highlights that this framework remains hypothesis-driven and requires direct experimental validation, particularly in hepatic models.

## Introduction

Sedentary lifestyles and high-fat, high-salt diets significantly increase the risk of metabolic dysfunction-associated steatotic liver disease (MASLD), a major global public health concern (Riazi et al., 2022; Younossi et al., 2023). According to the 2022 *Lancet Gastroenterology & Hepatology* Commission report, the global prevalence of MASLD was approximately 25% and is projected to increase to 33.5% by 2030 (Huang et al., 2021; Karlsen et al., 2022). Data from *China Liver Disease Epidemiology and Disease Burden White Paper* (2025) indicate that, as of 2022, approximately 210 million of the 400 million individuals with chronic liver disease in China had MASLD (Chan et al., 2023; Hutchison et al., 2023).

MASLD, previously referred to as nonalcoholic fatty liver disease (NAFLD) or metabolic dysfunction-associated fatty liver disease (MAFLD) in earlier literature, encompasses with a broad spectrum of clinical manifestations (Powell et al., 2021; Chan et al., 2023). As lipid accumulation progresses, MASLD can evolve from simple steatosis to inflammation and hepatocellular ballooning, eventually leading to hepatic architectural distortion. Approximately 30% of affected individuals may progress to metabolic dysfunction-associated steatohepatitis (MASH), fibrosis, cirrhosis, or even hepatocellular carcinoma (Friedman et al., 2018; Younossi et al., 2018; Powell et al., 2021; Rinella et al., 2024). Accordingly, elucidating the pathophysiology of MASLD is essential for effective prevention and treatment.

In response to stressors such as oxidative stress, viral infection, and aging, receptor-interacting protein kinases 1 and 3 (RIPK1 and RIPK3), together with mixed lineage kinase domain-like protein (MLKL), mediate necroptosis, a caspase-independent form of programmed cell death. Loss of plasma membrane integrity during necroptosis leads to the release of damage-associated molecular patterns (DAMPs), which further amplify inflammatory responses (Khoury et al., 2020; Yuan and Ofengeim, 2024). Increasing evidence indicates that necroptosis plays a critical role in MASLD pathogenesis (Afonso et al., 2015; Gautheron et al., 2015; Majdi et al., 2020; Xu et al., 2024). Clinical studies have shown elevated hepatic levels of the necroptosis-associated proteins, including phosphorylated MLKL (p-MLKL) and RIPK3, in patients with MASLD (Afonso et al., 2015; Gautheron et al., 2015)., with similar findings observed in animal models (Majdi et al., 2020; Li et al., 2025). For example, Li et al. (2025) showed that a 4-week high-fat diet (HFD) induced marked hepatic steatosis and vacuolation in rats, accompanied by elevated levels of total cholesterol (TC), low-density lipoprotein cholesterol (LDL-C), hepatic and serum triglycerides (TG), tumor necrosis factor-α (TNF-α), interleukin-6 (IL-6), RIPK1, RIPK3, and MLKL, as well as increased body and liver weights. Collectively, these findings suggest that necroptosis is a key component of MASLD pathogenesis; however, the precise mechanisms underlying its activation remain to be fully elucidated.

Currently, pharmacological treatment, dietary restriction, and exercise interventions are commonly used in the management of MASLD. Among these, exercise has emerged as a particularly effective non-pharmacological strategy. Clinical and experimental studies suggest that early exercise intervention-for example, 150 minutes of endurance training per week- can effectively prevent, delay, or even reverse hepatic steatosis while attenuating hepatic inflammation and fibrotic progression (Liu et al., 2024). To date, mechanistic studies on the effects of exercise in MASLD have primarily focused on apoptosis, pyroptosis, the mitochondrial unfolded protein response, and mitochondrial homeostasis, including mitophagy and mitochondrial dynamics (fusion/fission). However, whether exercise modulates necroptosis and its associated signaling pathways during MASLD progression remains unclear. To inform exercise-based therapeutic strategies, this review systematically synthesizes current evidence on MASLD, necroptosis, and exercise; clarifies the relationship between MASLD and necroptosis; and explores potential mechanisms by which exercise may prevent or ameliorate MASLD through the regulation of necroptosis.

## Necroptosis regulatory mechanisms

**Figure 1:**
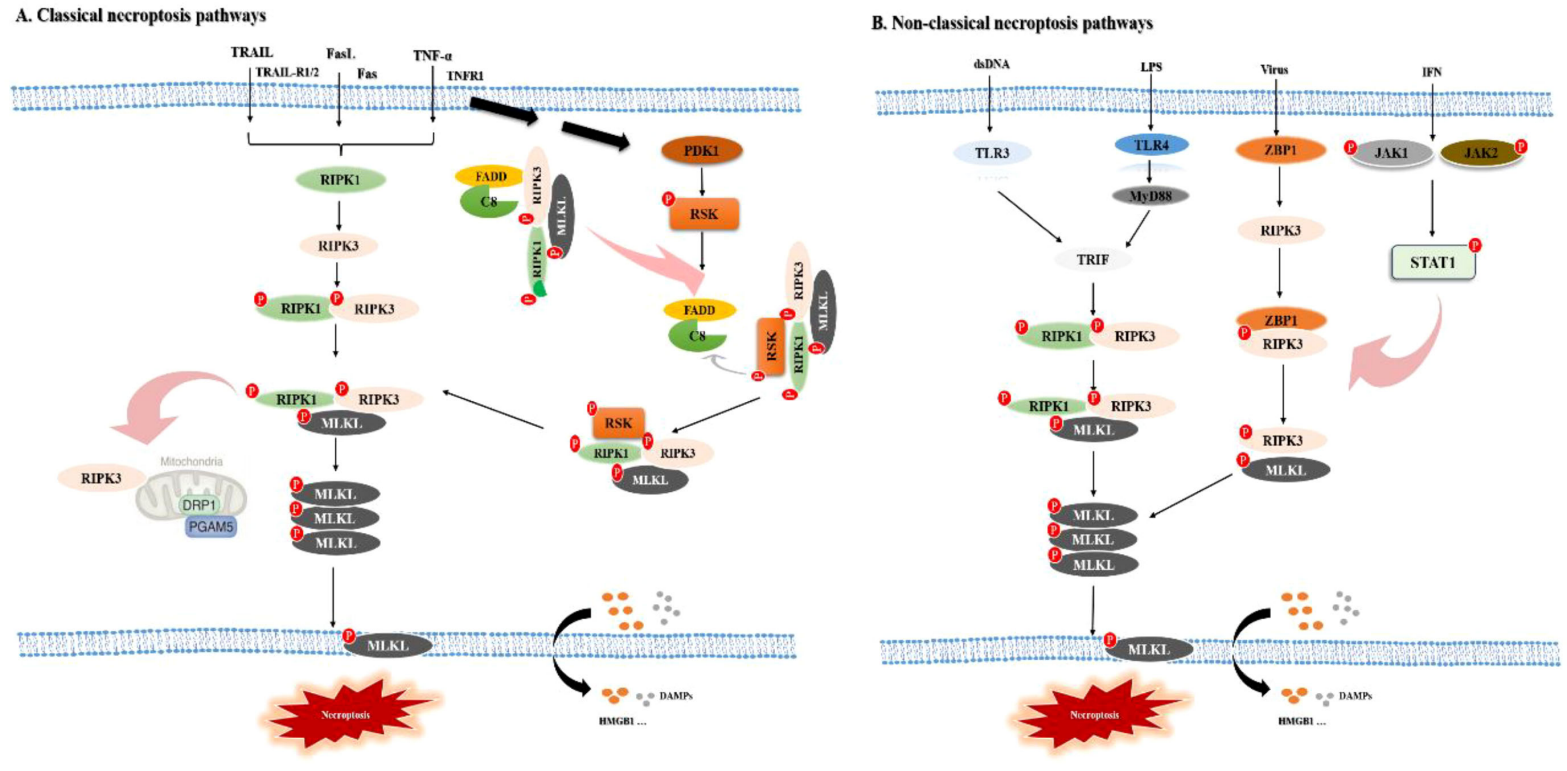
Molecular mechanism of necroptosis. A Classical necroptosis pathways, B Non-classical necroptosis pathways. **(A)** Classic necroptosis pathways, **(B)** Non-classical necroptosis pathways; tumor necrosis factor-related apoptosis-inducing ligand, TRAIL; Fas ligand, FasL; tumor necrosis factor, TNF-α; tumor necrosis factor receptor 1, TNFR1; receptor interacting protein kinase 1, RIPK1; receptor interacting protein kinase 3, RIPK3; mixed lineage kinase domain-like protein, MLKL; high mobility group protein B1, HMGB1; damaged-associated molecular patterns, DAMPs; phosphoglycerate mutase family member 5, PGAM5; dynamin-related protein 1, Drp1; 3-phosphoinositide-dependent protein kinase-1, PDK1; ribosomal S6 kinase, RSK); cysteine aspartate-specific protease 8, Caspase-8/C8; fas-associated death domain, FADD; double-stranded RNA, dsRNA; lipopolysaccharide, LPS; interferon, IFN; toll-like receptor 3, TLR3; toll-like receptor 4, TLR4; TIR-domain-containing adapter-inducing interferon-β, TRIF; myeloid differentiation primary response protein 88, MyD88; Z-DNA binding protein 1, ZBP1.

Necroptosis is regulated by both canonical and non-canonical signaling pathways (**Figure 1**). RIPK1 and RIPK3 are the principal mediators of the canonical pathway and activate downstream effectors such as phosphorylated MLKL (Ye et al., 2023; He et al., 2024). In contrast, upstream sensors and adaptor proteins, including Z-DNA binding protein 1 (ZBP1), Janus kinases 1 and 2 (JAK1/2), and Toll-like receptors 3 and 4 (TLR3/4), initiate non-canonical necroptosis signaling (Woznicki et al., 2021; Chen et al., 2022; Nakano et al., 2022).

### Classical necroptosis signaling

Death receptors like tumor necrosis factor receptor 1 (TNFR1), Fas (CD95), and TRAIL receptors commonly initiate the canonical necroptosis pathway, which is characterized by its dependence on RIPK1 kinase activity. Upon TNF-α binding to TNFR1, intracellular complexes such as complex I, complex IIa, and the necrosome are sequentially assembled (Xu and Huang, 2022). Complex I comprises TNFR-associated death domain (TRADD), TNFR-associated factor 2 (TRAF2), RIPK1, and cellular inhibitor of apoptosis protein 1/2 (cIAP1/2) (Wang et al., 2008; Amin et al., 2018; Lafont et al., 2018), whereas complex IIa consists of TRADD, RIPK1, fas-associated death domain (FADD), and cysteine-aspartate protease 8 (Casp8) (Tenev et al., 2011). Casp8 functions as a molecular switch by proteolytically inactivating RIPK1 and RIPK3 under homeostatic conditions. However, inhibition of Casp8 activity promotes RIP homotypic interaction motifs (RHIM)-dependent recruitment and activation of RIPK3 by RIPK1 (Sun and Wang, 2014; Seo et al., 2019). Activated RIPK3 subsequently phosphorylation of MLKL, including its oligomerization and translocation to the plasma membrane (Bedoui et al., 2020). There, oligomerized MLKL interacts with phosphatidylinositol phosphates to form membrane-permeabilizing pores. In addition to direct necrosome formation, TNF-α can induce necroptosis through mitochondrial pathways. Translocation of the necrosome to the mitochondria enables RIPK3-mediated phosphorylation of phosphoglycerate mutase family member 5 (PGAM5) on the outer mitochondrial membrane. PGAM5 then dephosphorylated dynamin-related protein 1 (Drp1) at Ser637, relieving its inhibition and promoting Drp1-mediated mitochondrial fission, loss of mitochondrial membrane potential, and necroptosis (Wang et al., 2012; He et al., 2024). Furthermore, an additional canonical pathway has recently involving the 3-phosphoinositide-dependent kinase 1/ribosomal S6 kinase (PDK1/RSK) axis has recently been identified. In this pathway, PDK1 promotes necroptosis signaling by activating RSK in an extracellular signal-regulated kinase (ERK)-independent manner (Yang et al., 2020).

### Non-classical necroptosis signaling

The non-canonical necroptosis pathway is primarily controlled by sensors such as ZBP1, TLR3/4, and interferons (IFNs) and does not dependent on RIPK1 kinase activity. ZBP1 recognizes viral Z-RNA via its Zα domains and recruits RIPK3 through its RHIM domain to initiate necroptosis (Jiao et al., 2020; Zhang et al., 2020). Toll/IL-1 receptor domain-containing adaptor inducing interferon-β (TRIF), a key adaptor in TLR3 and TLR4 signaling, promotes necroptosis by directly interacting with RIPK3 via its RHIM domain (Kaiser et al., 2013). IFNs stimulate necroptosis through both transcriptional and post-translational mechanisms. Activation of the JAK1/signal transducer and activator of transcription 1 (STAT1) pathway enhances the transcriptional program that facilitates RIPK1–RIPK3 complex formation (Nair et al., 2025). In addition, IFN-activated protein kinase R (PKR) can promote necrosome assembly through direct interaction with RIPK1 (Cao et al., 2024).

## Necroptosis and MASLD

Hepatocyte apoptosis, inflammatory cell infiltration, and excessive lipid accumulation in hepatocytes are the hallmarks of MASLD (Loomba et al., 2021; Chan et al., 2023; Hutchison et al., 2023; Xu et al., 2024; Ali and Lai, 2025). Hepatocyte death is a major driver of disease progression from simple steatosis to MASH, fibrosis, and ultimately hepatocellular carcinoma (Eslam et al., 2020; Golabi et al., 2023; Younossi et al., 2023; Lee et al., 2024). The “multiple-hit” model describing MASLD pathophysiology reflects complex interactions among oxidative stress, inflammatory signaling, metabolic abnormalities, and other factors (Friedman et al., 2018; Loomba et al., 2021). However, the exact causes of hepatocyte death remain incompletely understood. Emerging evidence indicates that necroptosis, a regulated and highly proinflammatory form of cell death, acts as a critical link between metabolic stress and hepatic inflammatory injury and likely contributes to the progression of MASLD (Xu et al., 2019; Sun et al., 2024).

Notably, RIPK1, RIPK3, and MLKL are significantly upregulated in the liver in MASLD, as observed in both clinical samples and experimental systems, and are positively associated with disease severity (Afonso et al., 2015; Gautheron et al., 2015; Roychowdhury et al., 2016; Xu et al., 2019; Briand et al., 2020; Majdi et al., 2020; Tao et al., 2020). For instance, mice with MASLD induced by 16–24 weeks of HFD feeding exhibit elevated serum alanine aminotransferase (ALT) levels, along with increased hepatic expression of RIPK1, RIPK3, and MLKL. Pharmacologic suppression of RIPK1 (e.g., utilizing RIPA-56) or genetic inactivation of RIPK1 significantly attenuates hepatic steatosis, inflammation, and fibrosis, accompanied by reductions in these markers (Majdi et al., 2020; Tao et al., 2020). Similarly, in the methionine-choline-deficient (MCD) dietary model, Ripk3 deletion reduces hepatic lipid accumulation and oxidative stress, Ripk3-deficient hepatocytes exhibit intrinsic resistance to necroptosis under basal circumstances (Roychowdhury et al., 2016). Interestingly, MASLD-like pathologies—including steatosis, fibrosis, and immune cell infiltration—can be induced in young mice by Ripk3 overexpression, whereas in aged mice, genetic deletion of Ripk3 or Mlkl markedly reduces inflammation, metabolic dysfunction, and spontaneous age-related liver injury (Mohammed et al., 2021; Mohammed et al., 2025). Taken together, these findings support a substantial role for necroptosis in MASLD pathogenesis.

Necroptosis is strongly induced by key inflammatory mediators in MASLD, including TNF-α (Cheng et al., 2015) and TLR4 signaling (Qian et al., 2022). Under conditions such as Casp8 inhibition, TNF-α binding to TNFR1 can trigger RIPK1/RIPK3-dependent necroptosis (Xu and Huang, 2022). Elevated hepatic and circulating levels of TNF-α and TNFR1 have been reported in both clinical and preclinical settings (Bansal and Bansal, 2024; Neira et al., 2024), and inhibition of TNFR1 attenuates liver injury, underscoring the pathogenic significance of this axis. Through the RHIM domain of its adaptor TRIF, which recruits RIPK3 to form a TRIF-RIPK3 necrosome, TLR4 can also directly induce necroptosis (Kaiser et al., 2013). Beyond executing cell death, necroptosis amplifies inflammation responses: dying hepatocytes release DAMPs such as IL-1α, high-mobility group box 1 (HMGB1) (Weinlich et al., 2017; Frank and Vince, 2019), which recruit inflammatory cells, activate Kupffer cells and other innate immune effectors, and stimulate hepatic stellate cells, thereby promoting fibrosis (Mohammed et al., 2021). Phosphorylated MLKL can translocate to mitochondria and increase mitochondrial membrane permeability, thereby promoting the cytoplasmic release of mitochondrial DNA (mtDNA). A positive feedback loop is established when cytosolic mtDNA activates the cyclic GMP-AMP synthase/stimulator of interferon genes/TANK-binding kinase 1 (cGAS/STING/TBK1) pathways, which subsequently induces type I interferons and other proinflammatory mediators (Ding et al., 2025). Oxidative stress, a key component of the multiple-hit model in MASLD, is bidirectionally linked to necroptosis. While lipid-induced mitochondrial or endoplasmic reticulum (ER) stress can indirectly activate the RIPK1/RIPK3 pathway (Weindel et al., 2022), oxidative modification of cysteine residues in RIPK1 can relieve its autoinhibition and directly activate necroptosis signaling (Zhang et al., 2017). Consequently, excessive necroptosis exacerbates fibrosis and hepatocellular injury, forming a self-sustaining pathogenic cycle (Xu et al., 2024). Targeting necroptosis-driven inflammation by preserving hepatic redox homeostasis while inhibiting RIPK3/MLKL signaling may therefore represent a promising therapeutic strategy for MASLD.

In conclusion, necroptosis is increasingly recognized as a central contributor to MASLD pathogenesis (**Table 1**). A self-sustaining pathogenic feedback loop arises when metabolic and inflammatory signals characteristic of MASLD activate the RIPK1-RIPK3 axis, which in turn propagates inflammatory and fibrogenic responses via DAMP release and cGAS-STING activation. Notably, emerging evidence indicates that RIPK1 and RIPK3 directly regulate hepatocyte lipid metabolism and impair mitochondrial function (Parisi et al., 2017; Parisi et al., 2019), thereby providing a mechanistic link between organelle stress and energy-metabolic dysregulation in MASLD progression. Collectively, these findings highlight the RIPK1–RIPK3 signaling axis as a potentially tractable therapeutic target in MASLD.

**Table 1:** MASLD and necroptosis.

Reference	Subjects	Experimental models	Key findings
Miyata et al (Miyata et al., 2021)	47~57-year-old MASLD participants	biopsy	p-MLKL^S358^↑
	5-week-old C57BL/6J mice, *Mlkl^-/-^* mice	HFD/Gao-binge model	p-MLKL^S358^↑, *p-Mlkl^S358^* mRNA↑
Afonso et al (Afonso et al., 2021)	29~74-year-old MASLD participants	biopsy	serum RIPK3↑, *Ripk3* mRNA↑
	7~8-week-old wild-type mice, *Ripk3*^-/-^mice	32-week/66-week MCD	liver weight/body weight→, fibrosis↓, *Tnf-α* mRNA*、Tlr4* mRNA*, Nlrp3* mRNA*, Cox-2* mRNA↓, p-MLKL^S345^↓; A positive correlation was observed between *Ripk3* mRNA and mRNAs for *TNF-α, Il-1β, Nlrp3, CD38, and CD68*.
Roychowdhury et al (Roychowdhury et al., 2016)	5-week-old male C57BL/6 mice, *Ripk3^−/−^* mice	12-week HFD	hepatic steatosis↑, ALT↑, inflammation↑, oxidative stress↑, *Ripk3* mRNA↑, RIPK3↑, p-MLKL↑
Saeed et al (Saeed et al., 2018)	8~9-week-old C57BL/6 wild-type mice、*Ripk3 KO* mice	12-week HFD	liver weight↑, body weight↑, liver TG↑, liver weight/body weight↑, AST→, TG→, ALT↑, *Ripk3* mRNA↓, *Pparα* mRNA→
Yang et al (Yang et al., 2019)	3~4-week-old male C57BL/6 mice	14-week HFD	serum ALT↑, serum TNF-α↑, RIPK1↑, RIPK3↑, p-MLKL↑,
Briand et al (Briand et al., 2020)	8-week-old male C57BL6/J mice	3-week HFD	body weight↓, hepatic steatosis↑, ALT↑, AST↑、TG↓, TC↑, FFA→, IL-10↑, INF-γ↑
Wandrer et al (Wandrer et al., 2020)	6~12-week-old C57/BL6J mice, TNFR1 knock-in mouse	24-week HFD	body weight↓, liver weight↓, hepatic steatosis↓, fibrosis↓, IR↓, ALT↓, Caspase-3↓, liver TC↓
Majdi et al (Majdi et al., 2020)	6-week-old male C57BL/6J mice	12-week HFD	RIPK1↑, RIPK3↑, MLKL↑, *Ripk1 mRNA*↑, *Ripk3 mRNA*↑, *Mlkl* mRNA↑, serum ALT↑
Wu et al (Wu et al., 2020)	5~6-week-old *Ripk3^-/-^*、*Ripk3^+/+^、Mlkl^+/+^*、*Mlkl^-/-^*mice	12-week HFD	MLKL↑, p-MLKL↑, MLKL oligomerization↑, *Tnf-α* mRNA↑*, Il-1β* mRNA↑*, Mcp-1* mRNA↑*, F4/80* mRNA↑
Tao et al (Tao et al., 2020)	C57BL/6J mice、*Ripk1^K45A/K45A^* mice	24-week HFD /5-week MCD	hepatic steatosis↓, serum ALT↓, liver TG↓, p-RIPK1^S166^↓, p-MLKL^S345^↓, *Tgfβ1* mRNA↓, *Col3a1* mRNA↓, *α-SMA* mRNA↓, Hyp↓
Amer et al (Amer et al., 2024)	male Wistar rats	5-day dexamethasone	body weight↓, ALT↑, AST↑, ALT↑, GGT↑, LDH↑, TC↑, TG↑, HDL-C↓, non-HDL-C↑, FBG↑, p-MLKL↑
Li et al (Li et al., 2025)	male SD rats	4-week HFD	body weight↑, liver weight↑, hepatic steatosis↑, vacuolization↑, inflammation↑, TG↑, TC↑, LDL↑, HDL↓, TC↑, TG↑, HDL↓, LDL↑, *Ripk1* mRNA, *Mlkl* mRNA, RIPK1↑, RIPK3↑, MLKL↑, TNF-α↑, IL-6↑, IL-4↓, IL-10↓

↑ Increase, ↓ Decrease, → No Change, FFA, free fatty acids; LDH, lactate dehydrogenase; HDL-C, high-density lipoprotein cholesterol; LDL-C, low-density lipoprotein cholesterol; FBG, fasting blood glucose; TG, triglycerides; TC, total cholesterol; LDL, low-density lipoprotein; HDL, high-density lipoprotein; ALT, alanine aminotransferase; AST, aspartate aminotransferase; IR, insulin resistance; MCD, methionine and choline deficient; INF-γ, interferon-γ; Hyp, hepatic hydroxyproline; CD38, cluster of differentiation 38; CD68, cluster of differentiation 68; NLRP3, NOD-like receptor protein 3.

## Exercise and necroptosis

### Acute exercise and necroptosis

At present, direct evidence linking acute exercise to necroptosis remains limited, and this section is based primarily on two studies from our group. Accordingly, the following findings should be interpreted as preliminary rather than conclusive. In 2023, our group reported that a single bout of downhill running (16 m/min, −16°, 90 min) modulated the expression of key necroptosis markers in gastrocnemius muscle. Both MLKL (total and phosphorylated at S358) and HMGB1 levels exhibited a biphasic pattern following exercise, peaking at 48 h and subsequently declining (Shi et al., 2023). Components of the ZBP1/RIPK3 pathway, including as ZBP1, RIPK3, p-RIPK3 S232, and the co-localization of ZBP1-RIPK3 and RIPK3-MLKL, were also transiently upregulated, with peaked levels observed at 48 h post-exercise. In a follow-up study conducted in 2024 using a similar exercise-induced muscle damage (EIMD) model, we found that MLKL (total and S358) was transiently increased in the soleus muscle, again peaking at 48 h. The expression of the DAMPs HMGB1 and IL-1α was displayed biphasic patterns, peaking at 48 h and 12 h, respectively. At 48 h after exercise, transmission electron microscopy revealed structural alterations in the soleus muscle, including incomplete and wrinkled double-membrane structures. Furthermore, the protein levels of RIPK1 and RIPK3 peaked at 48 h and remained elevated for up to 120 h (Ke et al., 2024). Their phosphorylation dynamic exhibited distinct temporal profiles: p-RIPK1 S166 showed a marked increase at 72 h following an initial rise and decline, whereas p-RIPK3 S232 was suppressed at 0 and 12 h and increased significantly only at 72 h (Ke et al., 2024). Although these studies suggest that acute exercise may activate necroptosis-related signaling in skeletal muscle via RIPK1/RIPK3- and ZBP1/RIPK3-dependent pathways, the current evidence remains insufficient to support generalized conclusions. Further independent studies are required to determine the reproducibility, their occurrence in other tissues, and whether they represent a general response to acute exercise or a context-specific effects of muscle-damaging protocols.

In conclusion, acute exercise may induce necroptosis with a distinct temporal pattern. Specifically, minimal changes are observed within the first 12 h post-exercise, followed by a progressive increase between 24 and 48 h, and a subsequent decline after 72 h. However, several important considerations should be noted. First, current evidence is largely restricted to skeletal muscle, and it remains unclear whether acute exercise induces necroptosis in other tissues or organs, such as the brain, liver, or heart. Second, the findings described above are derived from a single downhill running protocol (16 m/min, −16°, 90 min), and it remains unknown whether variations in exercise intensity, modality, or experimental model influence necroptosis-related responses.

### Chronic exercise and necroptosis

Apart from the impacts of acute exercise, chronic exercise has a major impact on the modulation of necroptosis. For example, Afousi et al. (2019) observed that the expression of necroptosis-related proteins (RIPK1, RIPK3, and MLKL) was considerably decreased in a rat model of myocardial infarction following 8 weeks of high-intensity interval training (HIIT). This decrease was linked to better cardiac function and antioxidant capacity, as well as lower lipid peroxidation and infarct size. Similarly, Fu et al. (2021) observed that the expressions of necroptosis-associated proteins (MLKL, p-CaMKII, p-RIPK3, p-RIPK1) and fibrosis markers (α-SMA, TGF-β) was markedly increased in two distinct atrial fibrillation mouse models—induced by either a 12-week HFD or a 3-week calcification-acetylcholine protocol. Interestingly, after four weeks of moderate-intensity swimming, these pathological increases were significantly reduced. All of these results point to the possibility that long-term exercise may reduce inflammation and myocardial fibrosis, allowing for structural and functional cardiac remodeling, possibly through the inhibition of RIPK1/RIPK3-mediated necroptosis. Similar regulatory effects have also been observed in the brain. Leem et al. (2024) found that a four-week endurance exercise intervention in MPTP-treated Parkinson’s disease rats dramatically downregulated markers of necroptosis (MLKL, p-RIPK3), pathogenic α-synuclein forms, and inflammatory cytokines (IL-1β, TNF-α, iNOS). In parallel it increased the levels of key metabolic regulators (p-AMPK, SIRT3, FoxO3a) and cytoprotective and antioxidant proteins (MnSOD, NQO1, HO-1, Nrf2) (Leem et al., 2024). These changes imply that endurance training reduces oxidative stress and neuroinflammation, at least partially, by suppressing necroptosis signaling.

In conclusion, both acute and long-term exercise can influence necroptosis (**Table 2**), although the specific effects appear to be highly tissue-dependent and time-dependent. Available studies suggest that long-term exercise training is generally associated with reduced necroptosis-related signaling in several tissues, whereas acute exercise more often induces transient and dynamic changes in necroptosis-related markers. In some contexts, such regulation may contribute to tissue homeostasis by reducing inflammation or oxidative stress. However, it should be clearly acknowledged that all studies summarized in **Table 2** were conducted in skeletal muscle, cardiac tissue, or brain tissue, and none were performed in the liver. Given that the regulation of necroptosis in hepatocytes and Kupffer cells may differ fundamentally from that in these other tissues, these findings cannot be directly extrapolated to the liver, nor can they be considered direct evidence that exercise regulates hepatic necroptosis to improve MASLD. Therefore, this represents a major limitation of the current evidence base, underscoring the urgent need for direct studies in hepatic tissue, particularly in MASLD-related models, to determine whether similar exercise-induced regulation of necroptosis occurs in the liver.

**Table 2:** Exercise and necroptosis.

References	Subjects	Tissues	Interventions	Key findings
Shi et al (Shi et al., 2023)	8-week-old male SD rats	Gastrocnemius Muscle	a bout of downhill running 16m/s, ─16°, 90min	ZBP1(48h/72h)↑, RIPK3(24h/48h/72h)↑, p-RIPK3^S232^(12h/24h)↑, MLKL(48h/72h)↑, p-MLKL^S358^(48h)↑, HMGB1(12h/24h/48h)↑
Ke et al (Ke et al., 2024)	8-week-old male SD rats	Soleus Muscle	a bout of downhill running 16m/s, ─16°, 90min	p-MLKL^S358^(48h/72h)↑, MLKL(24h/48h)↑, HMGB1(24h/48h)↑, IL-1α(12h/24h/48h)↑, RIPK1(0h~120h)↑, p-RIPK1^S166^(48h)↑, p-RIPK1^S166^/RIPK1(72h↑/120h↓), RIPK3(48h~120h)↑, p-RIPK3^S232^(0h/12h↓, 48h↑), p-RIPK3^S232^/RIPK3(12h/48h↓)
Afousi et al (Afousi et al., 2019)	6-week-old male Wistar rats	Myocardium	8-week HIIT 40min/session, 25°, 3 times/week	RIPK1↓, RIPK3↓, MLKL↓
Fu et al (Fu et al., 2021)	8~10-week-old male C57BL/6J mice	Myocardium	4-week moderate-intensity swimming 60min/day, 7 session/week	MLKL↓, p-CaMKII↓, CaMKII↓, p-CaMKII/CaMKII↓, RIPK3↓, p-RIPK3↓, p-RIPK3/RIPK3→, RIPK1→, p-RIPK1↓, p-RIPK1/RIPK1↓
Leem et al (Leem et al., 2024)	7-week-old male C57BL/6 mice	Brain	4-week rotarod walking exercise 8~16rpm, 20~38min/day, 5 day/week	MLKL↓, p-RIPK3↓, p-RIPK1↓, α-syn↓, p-α-syn↓, pro-IL-1β↓, IL-1β↓, TNF-α↓, iNOS↓, p-IκBα/t-IκBα↓, MnSOD↑, NQO1↑, HO-1↑, p-AMPK↑, Nrf2↑, SIRT3↑, FoxO3a↑
Zhou et al (Zhou et al., 2025)	22~28-year-old young soldiers	Saliva	4-week HIIT	Necroptosis↓

Increase, ↓ Decrease, → No Change, α-syn, α-synuclein, SD, Sprague-Dawley; iNOS, inducible nitric oxide synthase; IκBα, inhibitor of nuclear factor kappa-B α; MnSOD, manganese superoxide dismutase; NQO1, NAD(P)H:quinone oxidoreductase 1; HO-1, heme oxygenase-1; AMPK, AMP-activated protein kinase; Nrf2, nuclear factor erythroid 2-related factor 2; SIRT3, sirtuin 3; FoxO3a, forkhead box O3; HIF-1, hypoxia-inducible factor 1.

## The role of necroptosis in MASLD prevention and exercise-based therapy

As mentioned earlier, one of the pathogenic processes driving MASLD is necroptosis (Sun et al., 2024; Xu et al., 2024). One effective strategy for MASLD treatment and prevention is exercise; it improves insulin resistance, increases hepatic lipid catabolism, and reduces metabolic abnormalities linked to MASLD (Zhu et al., 2022; Chen et al., 2023; Rong et al., 2023) (**Table 3**). However, it is still unclear whether exercise affects MASLD by modifying necroptosis. A thorough analysis of the potential mechanisms will focuses on the RIPK1/RIPK3, the AMPK/mammalian target of rapamycin (mTOR), and the cGAS/STING pathway.

**Table 3:** Exercise and MASLD.

References	Subjects	Interventions	Key findings
Piguet et al (Piguet et al., 2019)	8~9-week-old C57BL/6 mice, *Pten^KO^* mice, *Fxr*^-/-^ mice	acute exercise 15min, 3h	15min *Prkaa2*↑, *Irs2*↑, *Pik3r1*↑, *Kank1*↓, *Ptprf*↓, *Itgb1*↑*、Zeb2*↑, *Spred2*↑, *Map 3 k3*↑, *Daam1*↑, *Il6ra*↓, *Il15ra*↓, *Socs2*↑, *Stat5a*↓, 3h *Cdkn1a*↑, *Smyd2*↓, *Prkaa2*↓, *Ddit4*↑, *Srebf1*↓, *Sox9*↑, *P2ry1*↓
Risikesan et al (Risikesan et al., 2023)	40~70-year-old male obesity MASLD subjects	acute exercise 90min, 50%VO_2peak_	Myocardial FFA kinetics (oxidation, uptake, esterification)↑, Hepatic FFA kinetics (oxidation, uptake, esterification)↓, VDL-TG↓, blood lactate↓
Gehrke et al (Gehrke et al., 2019)	male C57BL/6J mice	4-week endurance exercise	body weight↓, liver weight↓, fibrosis↓, ballooning↓, TG→, CCL2↓, AMPKα↑, p-AMPKα/AMPKα↑, p-AKT↑, p-AKT/AKT↑, NF-κB p65↓, *Pck1* mRNA→, *G6pc* mRNA→, *Fbp1* mRNA↓, *Il-6* mRNA↓, *Il-1β* mRNA→, *Ccl2* mRNA→, *Tgf-β* mRNA→, *Adgre1* mRNA↓
Huber et al (Huber et al., 2019)	24~61-year-old male MASLD participates	8-week endurance + resistance training 3~5 times/w	VO_2peak_↑, ALT↓, AST↓, hsCRP↓, ferritin↓, CK18↓, FLI↓, FIB-4↓, APRI↓, PRO-C3↓, C4M2↑
Abdelbasset et al (Abdelbasset et al., 2019)	45~60-year-old diabetic obesity MASLD subjects	8-week HIIT 3d/w, 40min/session, 80%~85%VO_2max_	BMI↓, liver TG↓, TC↓, HDL↓, LDL↓, VDL↓, HbA1c↓, HOMA-IR↓, ALT↓, FBG↓, VO_2peak_↑
Maillard et al (Maillard et al., 2019)	male Zucker obese rats	10-week HIIT 5d/w, 6 times/session	inflammation↓, FBG↓, total fat content↓, epididymal fat content↓
		10-week endurance exercise 5days/week, 12m/min, 51min/session	inflammation↓, FBG↓
Abdelbasset et al (Abdelbasset et al., 2020)	40~60-year-old diabetic obese subjects	8-week HIIT 3d/w, 40~50min/session, 80~85%VO_2max_ 8-week endurance exercise 60~70%HR_max_, 40~50min/session, 3times/w	liver fat↓, visceral fat↓, intergroup liver fat and visceral fat→
Fredrickson et al (Fredrickson et al., 2021)	5-week--old C57BL/6J mice	16-week HIIT 3d/w, 18~25m/min, 60min/session 16-week endurance exercise 3d/w, 13m/min~20m/min	body weight↓, fat mass↓, lean body mass↓, liver weight↓, TG↓, ALT↓, AST↓, *Mcp1* mRNA↓*, Sele* mRNA↓*, Icam1* mRNA↓*, Il1β* mRNA↓*, Tnf-α* mRNA↓*, Inos* mRNA↓, *Col1α1* mRNA↓, *Timp1* mRNA↓, *Mmp2* mRNA↓, *Tgf-β1* mRNA↓, *Actα2* mRNA→, Collagen↓, α-SMA↓
Iraji et al (Iraji et al., 2021)	10~15-year-old male adolescents obesity MASLD subjects	8-week HIIT 3d/w, 1~2w, 2 groups×6×30s/time, 36min/d;3~5w, 2group×7×30s/time, 38min/d, 6~8w, 2 group×8×30s/time, 40min/d	BMI↓, waist-to-hip ratio↓, body fat percentage↓, VO_2_peak↑, IR↓, TG↓, TC↓, ALT↓, AST↓, HDL↓, LDL↓
Bae (2020)	4-week-old C57BL/6J mice	8-week resistance exercise 5d/w, 50%~65%-70% load 8-week endurance exercise 5d/w, 50min/d	lipid degeneration↓, fat content↓, TG↓, p-AMPK↑, p-AMPK/AMPK↑, CB1→, AMPK→, CPT1↑, SREBP-1c↓
O’Gorman et al (O'Gorman et al., 2020)	45~77-year-old MASLD subjects	12-week endurance exercise 3~5d/w	hepatocyte swelling↓, fibrosis↓, steatosis→, lobular inflammation→
Takahashi et al (Takahashi et al., 2020)	36~70-year-old MASLD subjects	12-week resistance exercise 20~30min/time, 3 sets of push-ups + 3 sets of squats	body weight→, BMI→, ALT↓, CK18↓, FGF21↓
Charatcharoenwitthaya et al (Charatcharoenwitthaya et al., 2021)	35~40-year-old MASLD subjects	12-week endurance exercise 60min/d, 5d/w, 60%~70% HRmax, 12-week resistance exercise 60min/time, 60% 1RM	liver fat content↓, body weight↓, total body fat↓, fatty degeneration↓
Waters et al (Waters et al., 2022)	65~75-year-old obese elderly subjects	26-week endurance exercise 3d/w, 60-90min/time、 26-week resistance exercise 8~12 time/group, 2~3group/time, 85% 1RM 26-week endurance + resistance exercise	body weight↓, subcutaneous fat↓, visceral fat↓, intermuscular fat in thighs↓, insulin sensitivity↓
Stine et al (Stine et al., 2022)	18~69-year-old male MASLD subjects	20-week endurance exercise 5d/w, 30min/session	PAI-1↑, VO_2peak_↑, PDFF↑
Babu et al (Babu et al., 2022)	18~70-year-old MASLD subjects	12-week HIIT 2 d/w, 40min/session	FBG↓, VO_2max_↑, BMI→, body weight→, serum ALT→, AST→, TG→
Yu et al (Yu et al., 2019)	8~10-week-old female SD rats	4-week endurance exercise 30 min/d, 6d/w	body weight↓, fat mass↓, FFA↓, ALT↓, TG↓, INS↑, NF-κB↓, IL-6↓、TNF-α↓, MDA→, Nrf2↑, Keap1↓, SOD↑, IκBα↑, *Pparα mRNA*↓, *Chrebp mRNA*↓, *Fsp27 mRNA*↓, *Lxr mRNA*↓, TRIM72↓, p-IRS1^sec307^↑, PI3K↑, p-AKT^T485^↑, p-mTOR^Ser2448^↑, GLUT4↑
Tang et al (Tang et al., 2019)	6-week-old C57/BL6 mice, MIF*^KO^* mice	10-week endurance exercise 5d/w, 60min/session	body weight↓, TC↓, TG↓, steatosis↓, MIF↓, *Mif* mRNA↓, *miR451* mRNA↓, p-AKT/AKT↓, p-GSK3β/t- GSK3β↓
Rome et al (Rome et al., 2022)	Liver *Pck1^KO^* mice, wild-type mice	acute exercise 30min	INS→, FBG↓
		6-week endurance exercise 5d/w, 60min/time	body weight↓, BFP↓, TG↓, muscle glycogen↑, TKB↓, CS↑, ETC complexes↑, SCOT↑, BDH1↑
Keating et al (Keating et al., 2023)	46~66-year-old male obesity subjects	12-week HIIT 3d/w, 85~95 HRmax	IS↑, exercise capacity↑
Souza-Tavares et al (Souza-Tavares et al., 2024)	male C57BL/6J mice	10-week HIIT 3d/w, 12min/session	body weight↓, steatosis↓, TG↓, serum leptin↓, BG↓, NIS↓, *Gadd45* mRNA↓, *Atf4* mRNA↓, *Chop* mRNA↓, *Xbp1* mRNA↓, *Ppar*γ mRNA↓, *Srebp1c* mRNA↓, *Pparα* mRNA↑, *Fgf21* mRNA, *Sirt1* mRNA↑, *Pgc1α* mRNA↑, *Irisin* mRNA↑, *Pparγ* mRNA*/Pparα* mRNA↓, ACOX1↑, GRP78↑
Harris et al (Harris et al., 2024)	25~69-year-old subjects	20-week endurance exercise 5d/w, 30min/session	ALT↓, CK18↑, FPG↓, HbA1c↓, PDFF↓, liver volume↓
Zhu et al (Zhu et al., 2021)	7-week-old male C57BL/6 mice	8-week endurance exercise 5d/w, 12~20m/min, 20~50min/time	body weight↓, liver weight↓, serum ALT↓, AST↓, fibrosis↓, steatosis↓, lobular inflammation↓, ballooning↓, FABP4↓, TGFβ1↓, COL1↓, IL-1β↓, IL-6↓, *Fabp4* mRNA↓, *Ppara* mRNA↑, *Tgf-β1* mRNA↓, *Col-1* mRNA↓
Hu et al (Hu et al., 2023)	6~8-week-old C57BL/6 mice	8-week HIIT 5d/w, 8.8~11.6m/min, 12 times/session 8-week endurance exercise 5d/w, 14m/min, 60min/d	body weight↓, fat mass↓, GTT↓, IS↓, TC↓, TG↓, cGAS→, STING↑, p-TBK1↑, p-IRF3/IRF3↑, IL-6↓, IFN-β↓
Luo et al (Luo et al., 2024)	8-week-old female C57BL/6J mice, *Klf10^LKO^* mice, *Klf10^LTG^* mice	8-week endurance exercise 6d/w, 60min/d, 12 + 2m/min/w	AST↓, ALT↓, MDA↓, liver TC, TG↓, DAG↓, SCD-1↓, Collagen-І↓, Cleaved-Caspase-3↓, *Col1a1* mRNA↓, *Mmp13* mRNA↓, *Tgf-β1* mRNA↓
Chen et al (Chen et al., 2025)	6-week-old male C57BL/6J mice、*Erbb4^LKO^* mice、*Erbb4^flox/flox^* mice	8-week endurance exercise 6d/w	AST↓, ALT↓, TG↓, TC↓, *IL-6* mRNA, *IFNβ1 mRNA*↓, *TNFα mRNA*↓, *Scd1 mRNA*↓, *Fasn mRNA*↓, *Srebf1 mRNA*↓
Sardar et al (Sardar et al., 2025)	6~8-week-old male Wistar rats	8-week HIIT 5d/w, 30min/session, 10~15m/min, 80~95%VO_2max_ 8-week endurance exercise 5d/w, 60min/session, 40~60% VO_2max_	body weight↓, IR↓, ALT↓, AST↓, *Gdf15* mRNA↑, *Foxo1* mRNA↑, *Akt2* mRNA↑, *N-fκb* mRNA↓, *Tnf-α* mRNA↓, *Il1β* mRNA↓
Zhang et al (Zhang et al., 2025)	8-week-old male C57BL/6J mice	8-week endurance exercise 5d/w	body weight↓, liver weight↓, TG↓, TC↓, LDL-C↓, HDL-C↑, ACOX1↓, HMGCS2↓, CPT-1α↑,

FFA, free fatty acids; BMI, body mass index; IR, insulin resistance; HIIT, high-intensity interval training; HbA1c, glycohemoglobin; VO_2peak_, peak oxygen uptake; VDL, very low-density lipoprotein; VDL-TG, very low density lipoprotein triglyceride; CK18, cytokeratin 18; PAI-1, plasminogen activator inhibitor 1; PDFF, proton density fat fraction; FABP4, fatty acid-binding protein 4; TGFβ1, transforming growth factor-beta 1; COL1, collagen type I; PPARα, peroxisome proliferator-activated receptor alpha; SOD1, superoxide dismutase 1; Keap1, kelch-like ECH-associated protein 1; Nrf2, nuclear factor erythroid 2-related factor 2; MDA, malondialdehyde; IκBα, NF-κB inhibitor α; LXR, liver X receptor; ChREBP, carbohydrate-responsive element-binding protein; Fsp27, fat-specific protein 27; GLUT4, glucose transporter 4; TRIM72, tripartite motif-containing protein 72; IRS1, insulin receptor substrate 1; PI3K, phosphatidylinositol 3-kinase; AKT/PKB, akstrain transforming/protein kinase B; mTOR, mammalian target of rapamycin; AMPKα, AMP-activated protein kinase α; MIF, macrophage migration inhibitory factor; FLI, fatty liver index; FIB-4, fibrosis-4 calculator; APRI, AST to platelet ratio index; hsCRP, high-sensitivity C-reactive protein; PRO-C3, N-terminal propeptide of type III procollagen; C4M2, MMP-2-degraded type IV collagen neoepitope; PCK1, phosphoenolpyruvate carboxykinase 1; BDH1, β-Hydroxybutyrate Dehydrogenase 1; CS, citrate synthase; SCOT, succinyl-CoA:3-ketoacid coenzyme A transferase; GRP78, glucose-regulated protein 78; PPARγ, peroxisome proliferator-activated receptor γ; Chop, C/EBP homologous protein; FGF21, fibroblast growth factor 21; Gadd45, growth arrest and DNA damage-inducible 45; SREBP-1c, sterol regulatory element binding protein 1C; XBP1, X-box binding protein 1; ACOX1, acyl-coenzyme A oxidase 1; CB1, cannabinoid receptor type 1; IS, insulin sensitivity; GTT, glucose tolerance test; INS, serum insulin; BFP, body fat percentage; ETC, electron transport chain.

### RIPK1/RIPK3 pathway

The RIPK1/RIPK3 pathway is a one of the best-characterized necroptosis pathways. A RIPK1-RIPK3 complex is formed when RIPK1 and RIPK3 interact via their respective RHIM domains. This complex then activates downstream MLKL. Activated MLKL is then phosphorylated, oligomerizes, and disrupts plasma membrane integrity, thereby executing necroptosis (Sun and Wang, 2014; Seo et al., 2019; Bedoui et al., 2020). Current evidence indicates that RIPK1/RIPK3-mediated necroptosis is involved in the pathophysiology of MASLD. Chronic inflammation and oxidative stress are hallmarks of MASLD development (Royce et al., 2019), both of which can trigger the RIPK1/RIPK3 pathway (Kanazawa et al., 2022; Xu et al., 2024; Nair et al., 2025). The hepatic RIPK1/RIPK3 pathway is activated in animal models of HFD-induced MASLD/obesity, and either RIPK1 or RIPK3 inhibition reduces liver inflammation, fat accumulation, and fibrosis (Karunakaran et al., 2020; Majdi et al., 2020; Tao et al., 2020). Significant quantities of DAMPs (such as ATP and HMGB1) are released by hepatocytes going through necroptosis. Hepatic stellate cells (HSCs) and hepatic Kupffer cells are subsequently activated by these DAMPs, which promote the transformation of the HSCs into myofibroblasts and increase collagen production, ultimately resulting in liver fibrosis and perhaps cirrhosis (Yan et al., 2023).

By contrast, evidence linking exercise, necroptosis, and MASLD through this pathway remains indirect. As previously stated, exercise alters the structure and function of tissues by reducing necroptosis levels in the brain, myocardium, and other tissues or organs in AF and PD disease models through the RIPK1/RIPK3 pathway (Fu et al., 2021; Leem et al., 2024). Exercise is also well known to improve several pathological features of MASLD. However, to our knowledge, there is still no direct experimental evidence demonstrating that exercise alleviates MASLD specifically by regulating hepatic necroptosis through the RIPK1/RIPK3 pathway. Therefore, any proposed link between exercise and RIPK1/RIPK3-dependent necroptosis in MASLD should at present be regarded as a plausible but unproven mechanism. On the basis of the available evidence, it may be hypothesized that exercise could help limit hepatic lipid deposition and fibrotic progression partly by restraining excessive activation of the RIPK1/RIPK3 pathway, but this possibility requires further experimental validation.

Exercise may also improve MASLD by indirectly inhibiting necroptosis through a variety of mechanisms in addition to directly regulating the RIPK1/RIPK3 pathway. A well-known necroptosis inducer, TNF-α, is essential for RIPK1/RIPK3 pathway-dependent necroptosis. Exercise is related with lower levels of pro-inflammatory factors, such as TNF-α and IL-1β, in the liver and/or systemic circulation. Accordingly, it is reasonable to speculate that exercise may indirectly suppress RIPK1/RIPK3-mediated necroptotic signaling by lowering the levels of these inflammatory factors, although direct evidence in MASLD models is still lacking. Furthermore, Casp8 is important in the regulation of necroptosis. Under normal circumstances, Casp8, a pro-apoptotic protein, inactivates RIPK1 and RIPK3 via proteolysis. Reduced Casp8 activity, then triggers the RIPK1/RIPK3 pathway (Sun and Wang, 2014; Seo et al., 2019). Conversely, increased hepatic Casp8 activity can both induce apoptosis and prevent necroptosis. Some studies have reported that exercise can increase hepatic Casp8 activity, upregulate apoptosis-related molecules, and downregulate necrosis-related proteins. However, whether this shift directly alters the mode of hepatocyte death from necroptosis toward apoptosis in MASLD remains to be established. Thus, the possibility that exercise modulates the activation state of Casp8 and thereby influences RIPK1/RIPK3 signaling in MASLD should be considered a working hypothesis rather than a demonstrated mechanism.

In summary, current evidence supports a role for the RIPK1/RIPK3 pathway in MASLD pathogenesis, whereas the idea that exercise prevents or ameliorates MASLD by targeting this pathway remains largely inferential. Exercise may affect RIPK1/RIPK3 signaling either directly or indirectly through changes in inflammatory mediators such as TNF-α and regulators such as Casp8, but these proposed mechanisms have not yet been confirmed by direct experimental studies in MASLD.

### AMPK/mTOR pathway

AMPK serves as a cellular energy sensor, whereas mTOR complex 1 (mTORC1) is a master regulator of anabolism. AMPK can inhibit mTORC1 activity by phosphorylating Raptor and activating the tuberous sclerosis complex 2 (TSC2) (Szwed et al., 2021). Additionally, it promotes autophagy to eliminate damaged organelles by phosphorylating the unc-51-like autophagy-activating kinase 1 (ULK1) (Chun and Kim, 2021; Szwed et al., 2021; Ge et al., 2022). Because impaired autophagy, mitochondrial dysfunction, and metabolic stress are central features of MASLD, the AMPK/mTOR axis has been widely regarded as an important pathway linking energy sensing to hepatic lipid homeostasis and cell survival.

In addition to its role in the development and progression of MASLD, AMPK has recently been identified as a negative regulator of necroptosis (Wang et al., 2018; Lee et al., 2019; Wang et al., 2023). Previous mechanistic studies have shown that pharmacological activation of AMPK, for example with metformin or 5-aminoimidazole-4-carboxamide-1-β-D-ribofuranoside (AICAR), suppresses necroptosis, whereas inhibition or knockdown of AMPK enhances this process. The proposed mechanisms include regulation of the PGAM5 and Kelch-like ECH-associated protein 1 (Keap1) pathways, as well as promotion of pro-Caspase-8 (pro-Casp8) cleavage, may indirectly weaken RIPK1-RIPK3 complex formation (Wang et al., 2018; Wang et al., 2023). Furthermore, AMPK can influence the RIPK1-RIPK3 interaction and the necroptotic signaling by regulating Parkin-mediated mitophagy, thereby affecting mitochondrial function and ROS homeostasis (Lee et al., 2019). Collectively, these findings support the concept that AMPK may restrain necroptosis through coordinated suppression of mTORC1, enhancement of autophagic flux, and improvement of mitochondrial quality control.

A growing body of evidence indicates that exercise activates hepatic AMPK. Activated AMPK phosphorylates Raptor, inhibits mTORC1 activity, downregulates genes involved in protein and lipid synthesis, reduces hepatic lipid accumulation, and improves liver lipid metabolism and inflammation (Bae, 2020; Li et al., 2021; Bekheit et al., 2025). Based on these data, it is reasonable to propose that exercise may also suppress necroptosis by promoting autophagy, particularly mitophagy, through the AMPK/mTOR pathway. However, direct evidence for this mechanism in hepatic exercise models remains limited. Therefore, this relationship should be interpreted cautiously and framed as a plausible mechanistic hypothesis rather than a firmly established conclusion. According to recent findings, cysteine dioxygenase type 1 (Cdo1) is an upstream regulator of AMPK. Chen et al. (2023) reported that seven weeks of endurance training increased hepatic Cdo1 expression, which in turn interacts with and activates CaMKK2 and AMPK, thereby alleviating hepatic steatosis and promoting mitochondrial biogenesis and fatty acid oxidation. Importantly, the primary outcomes of that study were mitochondrial biogenesis and fatty acid oxidation rather than necroptosis-related markers. Therefore, the Cdo1-CaMKK2-AMPK axis currently supports the metabolic benefits of exercise in MASLD, but does not by itself directly demonstrate that exercise inhibits hepatic necroptosis through this pathway. At most, these findings provide indirect mechanistic support for a potential link, because enhanced AMPK activity, improved mitochondrial quality control, and increased fatty acid oxidation could theoretically create a cellular environment less permissive to RIPK1/RIPK3/MLKL-mediated necroptotic activation. Future studies should directly measure necroptosis-related endpoints, such as RIPK1, RIPK3, MLKL phosphorylation, or necrosome formation, in liver tissue from exercise intervention models to verify this hypothesis.

In summary, current evidence supports a clear role for exercise in activating the hepatic AMPK/mTOR pathway and improving steatosis, autophagy, mitochondrial function, and metabolic homeostasis in MASLD. In parallel, evidence from non-exercise or pharmacological studies indicates that AMPK activation can negatively regulate necroptosis. However, whether exercise suppresses hepatic necroptosis specifically through the AMPK/mTOR axis, including the Cdo1-CaMKK2-AMPK pathway, has not yet been directly demonstrated. Accordingly, this mechanism should be presented as a biologically plausible but still incompletely validated hypothesis. Targeted studies combining exercise interventions with genetic or pharmacological manipulation of AMPK/mTOR signaling and direct assessment of necroptosis will be necessary to establish causality.

### cGAS/STING pathway

When cytosolic DNA is detected, cGAS undergoes a conformational shift and catalyzes the formation of the second messenger, cyclic guanosine monophosphate-adenosine monophosphate (cGAMP) (Zhang et al., 2025). STING is subsequently bound by cGAMP and activated. TANK-binding kinase 1 (TBK1), NF-κB, and IFN regulatory factor 3 (IRF3) are among the downstream effector molecules that the activated STING recruits as it translocates from the endoplasmic reticulum (ER) to the Golgi apparatus (Dimitrov et al., 2025; Zhang et al., 2025). This cascade response ultimately results in the secretion and release of proinflammatory cytokines (such as TNF-α and IL-1β) and type I interferon (IFN-I) (Hu et al., 2022). According to recent research, the cGAS/STING pathway may have a major impact on necroptosis. For instance, when primary bone marrow-derived macrophages were treated with exogenous DNA in conjunction with the caspase inhibitor zVAD-fmk, Brault et al. (2018) noted a significant elevation of necroptosis and simultaneous activation of the cGAS/STING pathway. A significant rise in p-RIPK3 and p-MLKL expression confirmed this finding. Furthermore, increased permeability of the mitochondrial membrane induced by MLKL activation may lead to the release of mitochondrial DNA. The cGAS/STING pathway may then be activated by the released mitochondrial DNA, generating a positive feedback loop that exacerbates tissue damage and increases inflammation (Ding et al., 2025). Additional research shows that ZBP1 and MLKL expression is markedly increased in the absence of Casp8, which leads to activation of the cGAS/STING pathway. Moreover, ZBP1 activation can occur via reduced of Casp8 activity or direct activation of STING, consequently enhancing RIPK1/RIPK3-mediated necroptosis (Kelepouras et al., 2025). These findings suggest that the cGAS/STING pathway has a positive regulatory role in necroptosis.

Exercise can affect the progression of MASLD through modulation of the cGAS/STING pathway (Hu et al., 2023; Chen et al., 2025), although the currently available evidence is not entirely consistent. This inconsistency suggests that the response of the cGAS/STING pathway to exercise is highly context-dependent. Hu et al. (2023) established a MASLD mouse model that included a 7-week HFD followed by an 8-week HIIT intervention. They found that hepatic STING, p-TBK1, and the p-IRF3/IRF3 ratio were significantly decreased, but serum IL-6 and IFN-β levels were also significantly decreased. In contrast, Chen et al. (2025) exposed mice to endurance exercise for seven weeks following the induction of MASLD with a 16-week HFD, which caused cGAS activity to be phosphorylated and inhibited via activation of the AKT pathway. They discovered that exercise increased release of the adipokine neuregulin-4 (Nrg4) from adipose tissue. Nrg4 then activated the AKT pathway via its receptor Erb-B2 receptor tyrosine kinase 4 (Erbb4), leading to phosphorylates and inhibition of cGAS activity, thereby attenuating cGAS/STING-mediated inflammation and hepatic steatosis. Although these findings appear contradictory, the discrepancy is likely attributable to major differences in experimental conditions rather than being explained solely by “tissue-specific regulation.” First, exercise modality may be a critical determinant. HIIT imposes brief but intense and intermittent metabolic stress, which may transiently increase mitochondrial stress, DNA release, and innate immune signaling immediately after exercise, thereby resulting in short-term activation of hepatic cGAS/STING-related signaling. By contrast, endurance exercise more consistently promotes lipid oxidation, improves insulin sensitivity, and induces anti-inflammatory adaptations, which may favor suppression of cGAS activity through the adipose tissue–liver axis. Second, the duration of HFD exposure and the severity of MASLD may also shape the direction of pathway regulation. In the study by Hu et al., 7 weeks of HFD likely represents an earlier or less severe stage of metabolic injury, in which exercise-induced transient activation of the STING/TBK1/IRF3 axis may have contributed to adaptive stress responses, mitochondrial quality control, or metabolic remodeling, without necessarily causing sustained inflammation. In contrast, the 16-week HFD model used by Chen et al. more likely reflected a more advanced disease state characterized by greater lipotoxicity, chronic low-grade inflammation, and tissue injury; under such conditions, suppression of excessive cGAS/STING activation would be expected to confer greater benefit. Third, differences in the tissues and readouts examined should also be considered. Hu et al. primarily assessed signaling changes within the liver, whereas Chen et al. emphasized endocrine regulation originating from adipose tissue, highlighting a systemic liver-adipose tissue cross-talk mechanism. Moreover, despite the increase in hepatic STING-related signaling in the study by Hu et al., circulating inflammatory mediators such as IL-6 and IFN-β were reduced, suggesting that activation of proximal cGAS/STING signaling components does not necessarily translate into enhanced downstream inflammatory output. Exercise may reshape the magnitude, duration, and functional consequences of pathway activation rather than simply switching it on or off.

Taken together, the cGAS/STING pathway may play a biphasic or stage-dependent role in exercise-mediated regulation of MASLD. In the early stages of MASLD or under relatively mild metabolic stress, moderate and transient activation of hepatic cGAS/STING signaling may help maintain metabolic homeostasis, facilitate adaptive cellular responses, and promote the clearance of damaged organelles, thereby exerting beneficial effects. However, in the setting of prolonged HFD exposure, persistent lipotoxicity, and amplified inflammatory signaling, chronic overactivation of cGAS/STING is more likely to drive inflammation, cell death, and lipid metabolic dysfunction. In this latter context, exercise-induced inhibition of cGAS/STING—particularly through adipokine-mediated mechanisms such as the Nrg4-AKT axis—may be more effective in alleviating hepatic steatosis and chronic inflammation.

In light of the established regulatory role of cGAS/STING in necroptosis, it is reasonable to speculate that the beneficial effects of exercise on MASLD may depend on the fine-tuning of the “cGAS/STING-inflammation-necroptosis” axis, rather than on a simple unidirectional activation or inhibition of this pathway. Specifically, depending on exercise mode, intensity, intervention duration, and disease stage, exercise may either directly modulate hepatic cGAS/STING activity or indirectly suppress its pathological overactivation through anti-inflammatory adipokines such as Nrg4 released from adipose tissue. These effects may ultimately reduce necroptosis, improve lipid metabolism, and attenuate hepatic inflammation. Future studies should employ tissue-specific knockout or overexpression models to systematically compare different exercise modalities (e.g., HIIT, continuous endurance training, and resistance exercise), different stages of MASLD progression, and different sampling time points after exercise. Such studies are needed to clarify under which exercise conditions and disease contexts cGAS/STING activation represents an adaptive and beneficial response, and under which conditions it becomes pro-inflammatory and detrimental. This will help define the causal relationship within the exercise–cGAS/STING–necroptosis axis and provide new mechanistic insight for precision exercise-based prevention and treatment strategies for MASLD.

## Summary and perspectives

MASLD is a complex chronic metabolic disease, and accumulating evidence indicates that its onset and progression are closely associated with necroptosis. Current studies suggest that long-term physical exercise is generally associated with improved systemic metabolism, reduced inflammation, and downregulation of necroptosis-related signaling, whereas short-term or high-intensity acute exercise may induce stress responses in specific tissues, accompanied by dynamic changes in the expression of relevant proteins. However, it should be emphasized that the central proposition of this review—that exercise prevents or treats MASLD through regulation of necroptosis—is still based largely on indirect evidence and mechanistic inference, rather than on sufficient direct experimental evidence from hepatic exercise models. Therefore, while the protective effects of exercise against MASLD are well supported, whether and under which conditions these effects are mediated by modulation of necroptosis remains to be determined.

Available studies suggest that exercise may influence hepatic lipid metabolism, inflammatory responses, autophagy/mitophagy, and cell death processes through pathways such as RIPK1/RIPK3, AMPK/mTOR, and cGAS/STING. Nevertheless, in the context of exercise intervention in MASLD, the involvement of these pathways in regulating necroptosis should currently be regarded as a set of biologically plausible candidate mechanisms rather than fully established causal pathways. Accordingly, the proposed “exercise-necroptosis-MASLD” framework should be interpreted as a working hypothesis derived from integration of the available evidence.

Although this review systematically summarizes the potential mechanisms by which exercise may influence MASLD through regulation of necroptosis, several important issues still require further clarification: (1) Cell-type-specific variations: Further research is necessary, since various cell types (such as Kupffer cells and hepatocytes) may exhibit distinct necroptosis pathways. Future investigations should focus on clarifying the molecular pathways of exercise-induced necroptosis in the treatment of MASLD. (2) Impact of exercise modes: There is still limited direct evidence linking physical exercise, MASLD, and necroptosis. Basic questions include how different modalities (e.g., endurance vs. resistance, concentric vs. eccentric) differently regulate necroptosis, and whether exercise type and intensity affect necroptosis. (3) Interaction with other factors: It is crucial to investigate whether physical exercise may cooperatively regulate necroptosis and improve MASLD by interacting with other factors. It is expected that resolving these problems will provide new therapeutic targets for the exercise-centered management of MASLD.
